# A micro-costing study of mass-spectrometry based quantitative proteomics testing applied to the diagnostic pipeline of mitochondrial and other rare disorders

**DOI:** 10.1186/s13023-024-03462-w

**Published:** 2024-11-29

**Authors:** Francisco Santos Gonzalez, Daniella H. Hock, David R. Thorburn, Dylan Mordaunt, Nicholas A. Williamson, Ching-Seng Ang, David A. Stroud, John Christodoulou, Ilias Goranitis

**Affiliations:** 1 Economics of Genomics and Precision Medicine Unit, Centre for Health Policy, Melbourne School of Population and Global Health, University of Melbourne, 207-221 Bouverie St., Parkville, Melbourne, VIC 3010 Australia; 2 Murdoch Children’s Research Institute, Royal Children’s Hospital, Melbourne, VIC 3052 Australia; 3 Department of Biochemistry and Pharmacology, Bio21 Molecular Science and Biotechnology Institute, University of Melbourne, Parkville, VIC 3010 Australia; 4 Australian Genomics Health Alliance, Melbourne, VIC 3052 Australia; 5 Department of Paediatrics, University of Melbourne, Melbourne, VIC Australia; 6 Victorian Clinical Genetics Services, Murdoch Children’s Research Institute, Melbourne, VIC 3052 Australia; 7 Melbourne Mass Spectrometry and Proteomics Facility, Bio21 Molecular Science & Biotechnology Institute, The University of Melbourne, Melbourne, VIC 3010 Australia

**Keywords:** Proteomics, Mitochondrial disorders, Micro-costing, Functional genomics, Health economics

## Abstract

**Background:**

Mass spectrometry-based quantitative proteomics has a demonstrated utility in increasing the diagnostic yield of mitochondrial disorders (MDs) and other rare diseases. However, for this technology to be widely adopted in routine clinical practice, it is crucial to accurately estimate delivery costs. Resource use and unit costs required to undertake a proteomics test were measured and categorized into consumables, equipment, and labor. Unit costs were aggregated to obtain a total cost per patient, reported in 2023 Australian dollars (AUD). Probabilistic and deterministic sensitivity analysis were conducted to evaluate parameter uncertainty and identify key cost drivers.

**Results:**

The mean cost of a proteomics test was $897 (US$ 607) per patient (95% CI: $734-$1,111). Labor comprised 53% of the total costs. At $342 (US$ 228) per patient, liquid chromatography coupled tandem mass spectrometry (LC-MS/MS) was the most expensive non-salary component. An integrated analysis pipeline where all the standard analysis are performed automatically, as well as discounts or subsidized LC-MS/MS equipment or consumables can lower the cost per test.

**Conclusions:**

Proteomics testing provide a lower-cost option and wider application compared to respiratory chain enzymology for mitochondrial disorders and potentially other functional assays in Australia. Our analysis suggests that streamlining and automating workflows can reduce labor costs. Using PBMC samples may be a cheaper and more efficient alternative to generating fibroblasts, although their use has not been extensively tested yet. Use of fibroblasts could potentially lower costs when fibroblasts are already available by avoiding the expense of isolating PBMCs. A joint evaluation of the health and economic implications of proteomics is now needed to support its introduction to routine clinical care.

**Supplementary Information:**

The online version contains supplementary material available at 10.1186/s13023-024-03462-w.

## Background

Genomic sequencing has revolutionized diagnostics by detecting pathogenic variants in a single test, and thus ending diagnostic odyssey, avoiding unnecessary interventions, and restoring reproductive confidence for families [1–4]. One of the current challenges in the genomic diagnostics field, however, is assessing the functional impact of genetic variants in the approximately 80% of patients with rare disease suspected to have a genetic origin [5]. Hundreds to thousands of variants are typically identified following genome (GS) and exome (ES) sequencing, with a smaller number (sometimes tens to hundreds) of variants of uncertain significance (VUS) potentially needing follow up through functional assays [6]. Due to these challenges, approximately half of suspected rare disease patients remain undiagnosed despite undergoing GS or ES [7, 8].

Disease agnostic untargeted functional genomics approaches such as transcriptomics and proteomics have advantages over single biochemical tests for the upgrade of VUS as they can provide functional information, including the impact of VUS on gene expression, protein function, and gene-protein interactions for thousands of genes at once. We [9–15] and others [16, 17] have successfully applied proteomics to the detection of mitochondrial disorders caused by variants in genes encoding subunits, assembly factors, or other cellular machinery required for the function of mitochondrial respiratory chain (RC) complexes.

We recently demonstrated that proteomics can effectively replace RC enzymology (RCE) [15]. In Australia, RCE is the only functional test for mitochondrial disease (MD) that is offered as a National Association of Testing Authorities (NATA)-accredited clinical test. However the use of proteomics in this context avoids the use of invasive biopsies from tissues such as muscle, liver, and heart, provides greater sensitivity than RCE, and its untargeted nature means it has a high potential for use in other rare diseases where the affected gene is expressed in an available specimen.

As evidence of the effectiveness of functional genomics approaches emerges, the costs and cost-effectiveness of these technologies must be evaluated to inform their clinical implementation [18, 19]. To this extent, micro-costing studies of genomic diagnostic tests that [i] report detailed and complete procedures, [ii] provide transparency in the sources and methods to estimate resource use and unit costs, and [iii] that assess the uncertainty of their parameters, are necessary to provide a comprehensive evaluation of cost-effectiveness estimates [20]. This paper presents a micro-costing study conducted to estimate the economic cost of a Mass Spectrometry (MS) based quantitative proteomic diagnostic test for providing functional evidence to pathogenic variants in undiagnosed MD patients.

## Methods

### Study design

This study builds on the findings of our published systematic literature review of micro-costing genomics diagnostics [20], and aims to develop a micro-costing framework to estimate the cost of delivering proteomics diagnostic tests from a laboratory provider perspective.

### Setting and patient populations

This study collected resource utilization information to estimate the cost of an MS-based quantitative proteomics diagnostic test delivered by the Australian Undiagnosed Disease Network (UDN-Aus). The UDN-Aus is a collaborative project from a network of genetic clinicians and scientists in Australia that provides expert genetic testing services for individuals with rare disorders, and is a conduit for patient recruitment to various research programs that aim to increase the diagnostic rate for patients with undiagnosed genetic disorders. Such conditions include malformations of cortical development, epileptic encephalopathies, other presumed monogenic neurodevelopmental disorders and potential novel dysmorphic syndromes.

### Resource use identification

**Fig. 1:**
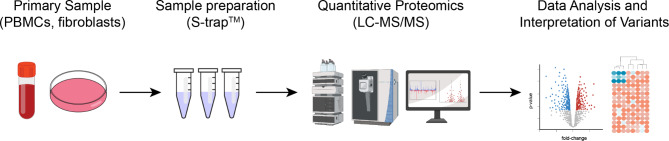
Proteomics analysis workflow. *Source*: RDMassSpec platform, Bio21 Institute

A workflow process (Fig. 1) for MS-based quantitative proteomics using cell-based samples such as blood derived peripheral blood mononuclear cells (PBMCs) or skin fibroblasts was developed in consultation with the project lead (DAS) and the lead research fellow (DH) of the RDMassSpec (Mass-Spectrometry based Functional Genomics Platform for solving Rare Genetic Disorders) platform. The workflow covered all steps of proteomics analysis from sample reception and isolation, protein volume measurement, mass spectrometry analysis, bioinformatics, and reporting. The main analysis focused on the costs of using PBMCs from EDTA blood samples. Similar to fibroblasts samples, PBMCs allow for the detection of over 5,000 intracellular proteins [21, 22], whereas fluids such as plasma (~ 500) [23], CSF (~ 1500) [24] or urine (~ 1,300 proteins) [25] are generally enriched in extracellular proteins and suffer extreme dynamic range. Resource utilization data were collected for each step of the workflow, including consumables (plasticware, reagents, buffers, protein assay kits), equipment (centrifuges, incubators, mass spectrometers), and labor (task time). A brief description of the clinical proteomics process, as well as a detailed summary of the resource units for consumables, equipment, and labor (Supplementary Tables 1–6) are provided in the supplementary materials. The costs of establishing fibroblast cell lines and for blood collection in the case of PBMCs analyses, including sample preparation, handling, processing, transport, and shipping are reported separately (Supplementary Table 7). Resource use in sampling was obtained at a UDN-Aus affiliated institution, and costs were based on agreed-upon pathology work orders from partner laboratories.

### Resource use measurement

Resource volumes for consumables and equipment in a 24-sample batch of proteomics tests were obtained through expert consultation. This batch size represents the maximum capacity for current operations. The batch includes 6 control samples and allows for processing 6 patients (requiring 3 sample replicates per patient). Time estimates for labor were provided by the laboratory personnel based on average processing times between February and June 2023.

PBMC isolation and the protein quantification (BCA) and digestion steps were conducted through sample batching; hence resource and labor estimates were derived for a 24-sample batch. On the other hand, the LC-MS/MS analytical steps were performed per sample, resulting in resource use and unit costs calculated on a per-sample basis and later reconciled with the batch quantity. A similar approach was taken for bioinformatics, reporting, and data archiving, which are performed at a per-patient level (3 replicates per patient), with labor and equipment use adjusted for the number of patients per batch.

Resource use for bioinformatics use was based on bioinformatician time and the use of high-performance computing equipment for LC-MS/MS data analysis. Bioinformatician time included maintenance, troubleshooting, automation tool development, ad-hoc analysis scripts, and data security management.

The estimates of labor use for the LC-MS/MS steps were obtained from the head (NW) and staff (C-SA) of the Mass Spectrometry and Proteomics Facility. The process uses the Orbitrap Exploris™ 480 mass spectrometer coupled with an UltiMate 3000 nanoHPLC platform (Thermo Fisher Scientific, Inc.). The labor estimates include time spent calibrating the equipment, loading gradients, running QC samples, instrument cleaning, and troubleshooting activities (Supplementary Table 2). For reporting activities, resource use was based on the time spent by laboratory personnel for report generation, review, dispatch to the genetics team and data archiving.

### Sample throughput and resource use valuation

An estimated maximum annual throughput of 986 rare disease patients (equivalent to 164 batches) was estimated. This estimate considered a 75% equipment utilization rate and it was used for all stages to reflect the processing capacity of the RDMassSpec platform. Approximately 2% of patient cases were expected to require re-analysis at the bioinformatics stage. Labor time for re-analysis was factored in based on the estimated proportion of cases requiring it.

To enhance transparency and replicability, published rates from manufacturers were used to determine the costs of consumables and equipment (Supplementary Tables 3–7). Consumable costs were calculated by multiplying mean per-unit costs by the number of units used in the testing process. In cases where only multiple use kit costs were available, the total cost was evenly distributed among all items in the package. Equipment costs were obtained by dividing the annual cost of each item by the estimated annual sample output, resulting in a cost per sample. The 75% equipment utilization rate was informed by previous micro-costing studies [26, 27], and it considered necessary maintenance and repair requirements.

Personnel costs were determined from the 2021–2025 Victorian public sector enterprise agreement for medical scientists, pharmacists, and psychologists (Victorian Hospitals’ Industrial Association, AG2022/4538). Stepwise costs were obtained by multiplying the estimated minute rate suitable for the grade of the staff performing the tests, by the equivalent number of minutes devoted to each task. For data archiving, a file size of 9 GB was estimated per patient, assuming 5-year storage. These costs were calculated based on the estimated file size and the cost per GB of digital storage from the University of Melbourne’s Compute and Data Infrastructure resource price estimator. The final cost estimates were calculated by multiplying the resource use estimates with their respective unit costs and then aggregating them to obtain the total cost estimate.

### Additional considerations

A 5 or 10 -year time horizon was chosen based on equipment lifetime estimates from the laboratory staff. Equipment acquisition costs were spread over the lifespan using straight-line depreciation and a 5% discount rate. Equipment sharing of pipettes, centrifuges and incubators was considered for the protein quantification and column digestion stages to reflect practice and avoid double costing of items. Maintenance costs for the LC-MS/MS equipment were based on manufacturer’s annual service contract rates. Overhead costs, including facility maintenance, cleaning, electricity, bulk gas and A/C utilization for the LC-MS/MS platform, were factored in by adding a 20% supplement to the total costs. A similar approach has been utilized in previous micro-costing studies of genomic sequencing [26, 28, 29] to account for overhead costs such as cleaning, facility administration costs and electricity.

### Analysis

All unit costs and the results of the analyses were estimated in 2023 Australian dollars (AUD$1 = USD$0.6682 as per the Reserve Bank of Australia’s (RBA) exchange rate in July 2023). A probabilistic analysis was conducted using 10,000 Monte Carlo simulations to capture the uncertainty of unit costs [30]. The costs of equipment, consumables and labor were collected as point estimates of mean use, with maximum/minimum value ranges provided where feasible. Each range was fitted to a gamma distribution via the method of moments [30] (Supplementary Table 8), and then used to draw 10,000 random values for each input using the *rgamma* function from the R Statistical Software (v4.1.2; R Core Team 2021). The mean value of the distribution served as the point estimate and a 95% confidence interval limit defined the upper and lower bounds.

One-way deterministic sensitivity analyses explored variations in key inputs and their impact on total costs. These included varying equipment utilization rates (50%-100), the overheads rate (10–30%), lower costs of consumables (-25%) and of the mass spectrometer and associated equipment (-25%), as well as different discount rates (1.5% and 5%), annual testing capacities (500 and 1,500 patients per annum), and the inclusion of sample reception costs, both for fibroblasts and PBMCs. A scenario analysis evaluated the variations in total costs at throughput levels between 100 and 1500 patients per year. Finally, a two-way sensitivity analysis evaluated joint changes in the annual throughput capacity alongside lower costs of the mass spectrometer or the bioinformatics processing time per patient.

### Exclusions

Costs related to start-up, general laboratory equipment (e.g., benchtops, fridges) pipeline development, quality control, training, and counselling before and after testing were excluded. Costs incurred by long term data storage, databanks, and ongoing research discovery were excluded as they are not typically included in hospital operating costs. In Australia, test procedures requiring research activities are ineligible for public funding [31].

## Results

The mean cost of a MS-based quantitative proteomic diagnostic test, including overheads, was $897 (95% CI: $734-$1252) [US $607 (95% CI: $485-$783)] per patient. Labor, consisting primarily of a technical scientist performing sample processing tasks as well as doctoral level scientists performing data analysis and reporting activities, was the main cost component at $396 (95% CI: $280-$560) [US $273 (95% CI: $188-$410)], or 53% of the total costs before overheads. Equipment costs, accounting for approximately 28% of costs or $209 (95% CI: $156-$269) [US $137 (95% CI: $96-$186)] per patient, were the secondary cost contributors. Consumables totaled $143 (95%CI = $111-$190) [US $96 (95% CI: $73-$131)], accounting for around 19% of the total costs before overheads. A detailed breakdown of costs per stage is presented in Table 1.

**Table 1:** Cost of proteomics testing per patient

Stage	Consumables	Equipment	Labor	Total (AU$)
PBMCs isolation	$45	$1	$8	$54 (35–93)
Protein quantification – Bicinchoninic acid (BCA) assay	$7	$2	$12	$21 (16–30)
Spin Column Digestion	$49	$2	$10	$62 (43–88)
Liquid Chromatography with tandem mass spectrometry (LC-MS/MS)	$42	$192	$109	$342 (253–482)
Bioinformatics	-	$4	$122	$126 (77–188)
Reporting	-	$0.3	$135	$135 (69–237)
Data archiving	-	$8	-	$8 (3–15)
Total cost excluding Overheads (95% CI)	$143 (111–191)	$209 (156–269)	$396 (280–560)	$748 (611–926)
Proportion of total cost	19%	28%	53%	-
Total cost including 20% overheads (95% CI)				**$897 ($734-$1**,**111)**

Most labor costs are attributed to the bioinformatics and reporting stages, where 215 min of labor time is dedicated to data analysis and reporting of activities. The LC-MS/MS stage was also a substantial contributor to labor costs, primarily driven by troubleshooting activities of variable complexity that require up to 12 h of labor time per case.

Around 62% of the equipment costs are derived from the acquisition and annual service/maintenance contract of the Orbitrap Exploris™ 480 MS platform ($135). High performance computing costs represented less than a dollar per patient ($0.60). These costs considered the acquisition of a dedicated 30-core computing system with the capacity of analyzing over 50,000 samples per year. The 5-year data archival and storage costs of $8 per patient (Table 1) were also notable contributors to equipment costs.

In the case of consumables, 34% of costs, or $49 per patient (Table 1), originated in the spin column digestion stage, which includes the use of S-Trap™ micro columns as well as plasticware (tubes and pipette tips). With an estimated cost of $45 per patient (Table 1), the PBMC isolation step represented 31% of consumable costs, with around $30 coming from the use of SepMate™-15 (IVD) columns and the Ficoll^®^ Paque Plus gradient. In addition, at $42 per patient (Table 1), the LC-MS/MS step was the third largest contributor to the cost of consumables, due to the Acclaim™ PepMap™ 100 analytical and trap columns ($30 per patient), which require a high replacement rate to comply with clinical laboratory accreditation standards.

### Sensitivity analysis

The results of the one-way deterministic sensitivity analysis are presented in Table 2. Reductions of 10% or 25% in data analysis and bioinformatician times led to a 2% and 4% fall in costs, respectively. Applying either a 10% or 30% rate for overheads resulted in a net change in costs of 8%; nonetheless, these changes did not impact on the costs of consumables, equipment, or labor. Using a 50% equipment utilization rate increased total costs by 14%, whilst a 100% utilization decreased costs by 6%, due to the impact of these changes in the per-sample cost of equipment. Discounting equipment costs at rates of 1.5% and 3.5% had a minor effect on total costs (Table 2). An annual output of 500 patients tested (around 50% of the estimated capacity) increased total costs by 32%. On the other hand, if annual testing reached the maximum estimated capacity of 1,500 patients for one LC-MS/MS platform, costs would fall by 11% (Table 2). Lowering the costs of consumables or the Orbitrap Exploris™ 480 MS and associated equipment by 25% reduced total costs by 4% and 5%, respectively. Finally, costs increased by 14% if blood sample reception costs were considered in the analysis, whereas a considerable 40% increase was obtained if fibroblasts samples are used, primarily due to the elevated costs of generating fibroblasts from skin biopsies.

**Table 2:** Results of the deterministic one-way sensitivity analysis

Parameter (base case value)	Variation	Cost (incl. overheads)	95% CI	% Change vs. base case
Base Case Analysis	-	$897.00	($734-$1111)	-
Data analysis & bioinformatician time − 98 min per patient	25% Less Bioinformatics Time	$859.00	($700-$1070)	-4%
	10% Less Bioinformatics Time	$883.00	($721-$1100)	-2%
Overheads 20%	Overheads 10%	$824.00	($674-$1021)	-8%
	Overheads 30%	$973.00	($794-$1211)	8%
Equipment Utilization 75%	50% Equipment Utilization	$1,020.00	($849-$1241)	14%
	100% Equipment Utilization	$840.00	($682-$1054)	-6%
Equipment discount rate − 5%	1.5% Equipment Discount Rate	$867.00	($707-$1087)	-3%
	3.5% Equipment Discount Rate	$883.00	($718-$1102)	-2%
Sample throughput − 986 patients per annum	500 (~ 50% less)	$1,185.00	($1003-$1413)	32%
	1500 (52% more - capping at 52% increase as it approaches the maximum annual capacity of one Orbitrap)	$796.00	($638-$1005)	-11%
Orbitrap Exploris™ 480 mass spectrometer – Cost of $980k AUD	25% Lower costs of the Orbitrap Exploris 480 MS	$861.00	($701-$1082)	-4%
Consumable costs	25% Lower Consumable Costs	$854.00	($695-$1065)	-5%
Use of PBMCs	Use of Fibroblasts (including line establishment and culture), and excluding PBMCs isolation costs from the workflow)	$1,428.00	($1216-$1680)	40%
Sample reception costs considered separately	Sample reception costs included	$1,020.00	($840-$1252)	14%

**Fig. 2:**
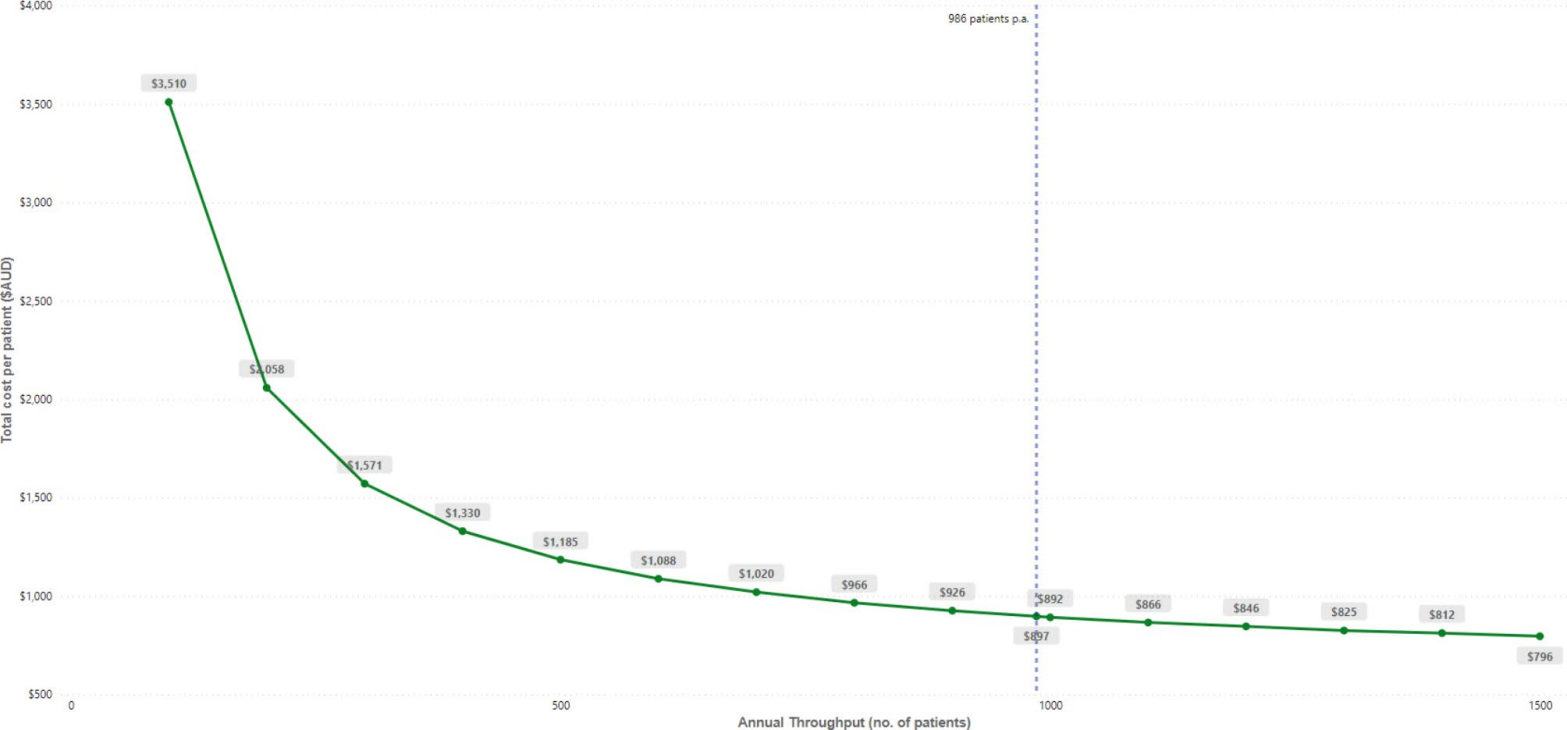
Scenario analysis. The plot displays the estimated costs per patient at throughput levels between 100 to 1500 patients per year. The dashed blue line presents the annual output estimated for the base case analysis (986 patients per annum)

The scenario analysis (Fig. 2) indicated that at a throughput of 100 patients per annum, the estimated cost per patient is $3,510 (95%CI = $3145-$3922), 291% higher than in the base- case. The figure goes down to $1,185 per patient (95%CI = $103-$1413) when the annual output reaches 500 patients per year. Total costs per patient go below $1,000 when the estimated annual output surpasses 800 patients, going from $966 (95%CI = $799-$1190) at an output of 800 patients, to $796 (95%CI = $638-$1005) at an annual throughput of 1,500 patients (Fig. 2). These figures represent reductions in costs per patient of 8% and 11%, respectively. A detailed presentation of all the results from the scenario analysis is included in Supplementary Table 9.

The results of the two-way sensitivity analysis (Supplementary Fig. 1 and Table 10) indicated slightly lower costs per patient at all throughput levels with 10% lower bioinformatics time. Reductions of 25% in the costs of LC-MS/MS equipment or consumables resulted in noticeable lower costs per patient at all throughput levels.

## Discussion

This study reports a detailed micro-costing analysis of Mass Spectrometry-based quantitative proteomics diagnostic tests for MDs and potentially other rare diseases. The workflow developed for our model presents the end to end costs from receipt of EDTA blood samples, to reporting and data archiving. The total cost of a test was estimated at $897 (US $607) per patient in the baseline calculations. Labor costs accounted for the largest portion (53%) of the total costs before overheads, followed by equipment and consumables. At $342 per patient (US $228), the LC-MS/MS stage was the major cost component, driven by the Orbitrap Exploris™ 480 MS and associated equipment costs.

Proteomics offers a practical alternative to targeted protein assays like western blotting and functional tests such as RCE in detecting primary mitochondrial disorders as it facilitates the measurement of multiple proteins encoded by subunits, assembly factors, or other cellular machinery necessary for the functioning of mitochondrial RC complexes. However, whilst MD is a type of rare disease that can be caused by mutation in over 350 genes [32–35], RCE is not gene specific as mutations in different genes can cause defects in the respiratory chain. Furthermore, RCE often involves invasive biopsies from tissues like muscle, liver, and heart, which require general anesthesia along with its costs and risks.

We have shown that proteomics is more sensitive for the detection of primary mitochondrial disorders in less invasive specimens such as skin derived fibroblasts or Epstein-Barr virus transformed lymphoblastoid cells from blood [7, 9–13, 36, 37]. For example, a study by Helman et al. (2021) [11] used integrated RNA sequencing and quantitative proteomic analysis to identify the underlying cause of disease in fibroblasts from two siblings after uninformative ES and GS. The multi-omic analysis confirmed a deep intronic variant in *NDUFB10* as the cause of the suspected MD. The increased sensitivity of proteomics over RCE was evident as proteomics could readily detect a specific mitochondrial defect in fibroblasts where RCE was uninformative. This is likely due to the statistical power arising from the measurement of multiple proteins representing each complex, underpinned by the quantification of multiple peptides. Moreover, the unbiased nature of proteomics and its emerging utility in secondary and non-mitochondrial disorders [11, 12, 38, 39], lends itself to unexpected findings, for example identification of mitochondrial phenocopies, and the dissection of complex structural rearrangements and copy number variants [40].

Integrating RNA sequencing and quantitative proteomic analysis has proven useful in identifying the causes of suspected MDs when ES and GS were uninformative [11]. This approach could potentially extend the diagnostic yield of MDs by 16% over GS or ES alone [6]. The potential diagnostic yield of proteomics alone under similar circumstances is not yet known, though in a study of 121 ES or GS unsolved patients suspected of MD, Kopajtich et al. (2021) [41] combined RNA sequencing with proteomics on fibroblasts, with a combined yield of 22%, while Hock et al. (2024) [15] used a panel of fibroblasts from genetically diagnosed MDs to demonstrate that proteomics could detect the specific defect in 88% of cases. The latter also benchmarked proteomics against RCE, which had a yield of 79%, but the untargeted nature of proteomics led to the additional diagnosis of several individuals that were GS or ES unsolved, including what turned out to be a non-mitochondrial disorder. Noting the time, inconvenience and expense needed to obtain skin fibroblasts, the authors also demonstrated the utility of PBMCs in providing functional proteomic evidence to enable variant upgrade with similar turnaround times to ultra-rapid genomic analysis. A recent study by Starosta et al. (2024) [42] also demonstrated the value of rapid proteomics in informing genomic analysis for critically ill infants. Rapid diagnosis can lead to substantial cost savings by reducing hospital stays, a key driver of costs for infants in critical care units [43–45].

The findings from this study provide valuable insights into the economic costs associated with proteomics diagnostics. The results from the base case analysis indicate that the cost of a proteomics diagnostic test is below the indicative price for respiratory chain enzymology (RCE) in Victoria of AUD $1130-$1525 (US $755-$1019) per tissue sample (Victorian Clinical Genetics Services, Parkville, Australia), noting that the cost of RCE does not include the additional costs associated with patient sample collection. In addition, our analysis indicates that the use of PBMCs is cheaper and more time effective than generation of fibroblasts, albeit not yet extensively tested [21, 22], with potentially lower costs when fibroblasts are readily available, as the costs involved in isolating PBMCs would be avoided. Furthermore, the presented workflow and estimates are reflective of resource use necessary for a proteomics test, making the model transferrable to emerging applications as the broader utility of proteomics in secondary and non-mitochondrial disorders is demonstrated.

Nevertheless, a high level of expertise is required to interpret the results accurately, which may limit the availability of MS-based quantitative proteomics diagnostic tests to organizations where highly skilled staff are available. This also represents high labor costs as presented in the current study. Furthermore, the potential of non-informative or inconclusive results [46], as well as the need for ongoing validation and standardization of the techniques present current challenges for widespread adoption.

In addition, like most biochemical tests used in the diagnosis of rare diseases, proteomics tests rely on blood or tissue samples, where not all genes of interest may be expressed. This may lead to negative/inconclusive results, needing multiple sample types from the patient or further investigations to confirm pathogenicity [6]. Furthermore, the accurate quantification of proteins at low levels of abundance remains a difficult task in quantitative proteomics analysis [47]. However, these activities have the possibility of being streamlined by implementing standardized protocols and utilizing automation technologies [47, 48]. As presented in the analysis, lower bioinformatics and data analysis times can cut labor costs and improve efficiency, resulting in reduced costs per patient.

Certain limitations are acknowledged in our study. The process workflow was developed in a research environment and may not be directly applicable to clinical settings. This was addressed by estimating resource use and utilizing salary scales and seniority levels for staff, that reflect the expected labor and process requirements, such as the rate of calibrating and replacing of equipment, under standardized and accredited procedures in a clinical diagnostics environment. The sample batching reflects current processing capacity and might not accurately represent forthcoming developments in technology and laboratory workflows. This was addressed by exploring the impact of multiple throughput levels on total costs in the sensitivity and scenario analysis. Similarly, the use of a 20% overheads rate was based on studies costing GS and ES and may underestimate the electricity, temperature control and gas supply requirements of MS-based proteomics analysis. However, rates of 10% and 30% for overhead costs were explored in the sensitivity analysis, with their impact on cost estimates presented in the results.

Finally, there are no established guidelines or standards to conduct micro-costing studies. We guided our approach using the findings of a recent systematic literature review to ensure transparency and rigor in our cost analysis [20], such as the identification and inclusion of all relevant cost components and clear reporting of data sources and assumptions. The study also incorporates parameter uncertainty through probabilistic analysis, which models the variability and fluctuations associated with the cost inputs, further enhancing the robustness of the results.

Our findings have potential implications for policymakers, given that federally funded GS and ES testing of rare disease patients was introduced in 2020 in Australia. Despite the lower than predicted utilization during the initial three years [49], service uptake is projected to rise following coordinated efforts to improve access. It is estimated that over 4,000 individuals in Australia undergo genomic testing each year due to rare diseases, which affect up to 2 million individuals in Australia [50]. Quantitative proteomics diagnostic tests have potential applications in secondary MD as well as non-MD monogenic diseases [12, 38, 39], offering the possibility of a diagnosis to the approximately half of rare disease patients whose cases remain unresolved after GS or ES. From the potential pool of 2,000 patients, not all patients would undergo additional functional testing due to a lack of clinical suspicion, use of alternative methods, or financial constraints. Under a conservative approach, we can estimate that between 100 and 500 of these patients would undergo proteomics testing per year, potentially increasing as the clinical benefit and funding pathways for the tests are established. Based on our analysis, this would represent a cost of $3,510 (US $2,345) per patient if the annual throughput is 100 patients, and $1,185 (US $792) per patient for an annual throughput of 500 patients tested. Furthermore, future growth in capacity presents potential reductions in costs, which can be further lowered through an integrated analysis pipeline and bulk discounts on equipment and consumable costs.

## Conclusions

Around 50% of individuals with suspected mitochondrial and other rare diseases remain undiagnosed, often requiring functional validation of variants identified by genomic sequencing approaches. At an estimated average cost of $897 (US $607) per test, untargeted proteomics is a potentially underutilized approach that can provide functional data on thousands of proteins in a single test to increase the diagnostic yield of rare diseases at a lower cost compared to current functional assays. An evaluation of the economic implications of the tests, changes in diagnostic outcomes, health-related quality of life as well as a valuation of their benefits are now needed to demonstrate its cost-effectiveness to inform their clinical implementation in Australia and worldwide.

## Electronic supplementary material

Below is the link to the electronic supplementary material.

Supplementary Material 1

## Data Availability

All data generated or analyzed during this study are included in this published article and its supplementary information files.
