# Supplementary material for: A micro-costing study of mass-spectrometry based quantitative proteomics testing applied to the diagnostic pipeline of mitochondrial and other rare disorders
Source: Orphanet J Rare Dis. 2024 Nov 29;19:443. doi: 10.1186/s13023-024-03462-w (PMC11605922; doi:10.1186/s13023-024-03462-w)
Supplement: Supplementary file 1 — Supplementary Material 1 [file 13023_2024_3462_MOESM1_ESM.pdf]

Supplementary Materials: Micro-costing proteomics diagnostic testing

This document presents detailed information on the proteomics process, as well as the assumptions, unit costs and associated sources, as well as the methodological considerations incorporated in the study. Additionally, the results of the scenario and the two-way sensitivity analysis are also presented.

PART 1: QUANTITATIVE MASS SPECTROMETRY AND DATA ANALYSIS

The clinical proteomics process starts with the receipt of full blood or primary fibroblasts samples. Choosing a cell-based sample such as PBMCs and skin fibroblasts allows for the detection of over 5,000 intracellular proteins (Hentschel et al., 2021; Yang et al., 2022), whereas fluids such as plasma (~500) (Bruderer et al., 2019), Cerebrospinal Fluid (~1500) (Barkovits et al., 2018) or urine (~1,300 proteins) (Muntel et al., 2015), are generally enriched in extracellular proteins and suffer extreme dynamic range. Thus, the use of a cell-based sample increases the chances of identifying the causative gene product among multiple candidates. Full blood samples are processed within 24h for isolation of peripheral blood mononuclear cells (PBMCs), while primary fibroblasts are typically cultured for two passages before sample preparation. Initially, fibroblasts or PBMCs pellets are lysed in a solution that consists of 5% SDS and 50 mM triethylammonium bicarbonate (TEAB) and total protein amount is measured using the Pierce BCA protein assay kit (Thermo Fisher Scientific). The same amount of protein is aliquoted for each sample which is then further reduced, alkylated and cleaned-up before overnight on-column tryptic digestion on S-trap™ micro spin columns (ProtiFi, LLC). Eluted peptides are dried down and then reconstituted for liquid Chromatography with tandem mass spectrometry (LC-MS/MS) analysis, where an enrichment column and analytical column are utilized to separate the peptides in a data-independent acquisition (DIA) mode. Analysis are performed with Spectronaut® software against referencing UniProt database, while R scripts and Perseus software are employed for data analysis tasks such as statistical tests and data visualization such as volcano plots.

Data reporting includes the quantification of the abundance level of thousands of peptides and proteins and statistical analysis between patient and control group in relation to the reported genetic variants and their predicted function based on analysis of current literature. In particular cases, data reporting can include detailed analysis of specific pathways and complexes or even peptide levels in cases of exon skipping or deletion and duplication events. This information could potentially explain the findings and support variant causation. Approximately 2% of patient cases were expected to require re-analysis at the bioinformatics stage when the analysis of a specific variant is requested by the genetics team, or after a negative result is delivered (when the analyzed protein is either not affected or it cannot be detected).

PART 2: ANALYTIC ASSUMPTIONS

Supplementary Table 1a: List of analytical assumptions for estimation of annual processing capacity

**Sample throughput**	**Maximum Output per year**: 5,256 samples<br>**Considering 100 minutes processing time per sample when the Orbitrap Exploris™ 480 Mass Spectrometer is running nonstop for 1 full year*
	**Estimated Output per year**: 3,942 samples<br>**Considering a 25% downtime (a 75% utilisation rate) to account for downtime for troubleshooting, cleaning and maintenance of the LC-MS/MS platform*.
	**Batches per year at current capacity:** 164 batches per year
	**Samples per batch**: 24 samples – Equivalent to 6 patients (3x samples per patient) and 6 controls
	**Estimated patients per year**: 986 patients per year

Supplementary Table 1b: List of analytical assumptions applied to measurement of sample obtention costs

**Sampling**	Blood Collection	All samples will incur these costs
	Sample handling and processing	All samples will incur these costs
	Sample Transport/Shipping	All samples will incur these costs

Supplementary Table 1C: List of analytical assumptions for measurement of resource use

**Consumables**	All estimates were calculated for a batch of 24 samples per run; this assumes the use of 5 controls and 3x repetitions per patient sample
	The protein quantification step (BCA) assumes the use of 9 standards; the standards are prepared in concentrated solutions that are stored in 5 Eppendorf

	tubes; the 5 concentrated solutions are assumed to last 15 runs based on the amount that's loaded into the wells (20µL)
	The protein quantification step (BCA) also assumes the use of 1x96-well BCA plate per run (as stated above, 1 run equals 24 samples)
	Consumables for the LC-MS/MS step were calculated as the quantity of resources needed to produce 14,000 samples, which was the estimated annual output in the facility during 2022
**Equipment**	Annual costs for equipment (including maintenance) were based on the yearly output of 3,942 samples for all other steps except for High Performance Computing (HPC).
	A 75% utilisation rate was assumed for all equipment (including software, high performance computing (HPC), except for data archiving, which is a cloud-based service.
	All equipment was assumed to have a 10-year lifespan, except for HPC and data archiving. Annual equipment costs (excluding the service maintenance) were calculated using a 5% discount rate over the lifespan using an annuity factor of 7.72. Service maintenance costs were discounted over a 6-year life span to reflect the duration of the manufacturer's service plan. HPC costs were discounted over a 5-year lifetime. Data archiving costs were not discounted as the inputs represent the yearly cost.
	HPC costs were calculated based on the acquisition of a platform with 32 cores and 128 GB RAM. The analysis software can use up to 30 cores of HPC for an analysis time of 30 minutes per patient, which yields an output of 39,420 samples per annum at a 75% utilisation rate.
	5-year storage was assumed for the data archiving step.
**Bioinformatics**	The bioinformatician is exclusive to the Proteomics lab; they devote 80-90% of their time to support the proteomics diagnostics project.
	Bioinformatician labour use was estimated using the midpoint of an 80-90% utilisation rate of an FTE employee (38 hours per week). Their total weekly time was divided by the estimated number of batches processed in a week to determine the number of minutes required per patient for maintenance activities. This yielded an estimated time of 387.6 minutes of labour time per batch, which was assigned to bioinformatics maintenance activities.
	There is one HPC exclusive to the lab (shared by all lab personnel). It has 32 cores and 128 GB of RAM.
	The re-analysis rate of patient cases was assumed to be 2% based on expert opinion.

PART 3: LC-MS/MS TROUBLESHOOTING ACTIVITIES

Approximately a quarter of samples require troubleshooting activities, which can vary greatly in processing time. To calculate labor use for troubleshooting, different processing time points (0 to 720 minutes) were factored in and weighted by the proportion of samples requiring each specific processing time point.

Rationale: 75% of samples don't require troubleshooting. Out of the 25% of samples that do need troubleshooting, rows 2-5 show the % of those samples that take each of the time values in the "Estimate" column to process. For example, the estimate in row 8 shows that 3% of samples take 4 hours of troubleshooting time.

Supplementary Table 2: Calculations of labor use for LC-MS/MS troubleshooting activities

Troubleshooting	Estimate	Unit	Time in mins	Percentage of samples	Weighted Percentage	Weighted Time (Mins)
N/A	0	mins	0	75%	75%	0.0
Min	5	mins	5	5%	1%	0.1
	15	mins	15	45%	11%	1.7
	30	mins	30	25%	6%	1.9
	1	hours	60	21%	5.3%	3.2
	4	hours	240	3.000%	0.7500%	1.8
Max	12	hours	720	1.000%	0.2500%	1.8
**Weighted processing time (in minutes) for troubleshooting (per sample)**						10.375

PART 4: RESOURCE USE MEASUREMENT

Supplementary Table 3: Labor – List of activities and associated labor use per stage

Stage	Step	Labor Time (minutes)	Personnel Type
**PBMCs isolation**[a]	SepMate & Ficoll assembly	6	Medical scientist
	Blood dilution & pipetting into SepMates	12	Medical scientist
	First spin	10	Medical scientist
	PBMCs layer collection	12	Medical scientist
	PBS wash spin	8	Medical scientist
	Discard supernatant + resuspend in 1ml PBS + transfer to Eppi tube	12	Medical scientist
	Second PBS wash spin	5	Medical scientist
	PBMCs pelleting & PBS removal	6	Medical scientist
**Protein quantification - Bicinchoninic acid (BCA) assay**[a]	Prepare diluted albumin (BSA) standards	5	Medical scientist
	Resuspend cell pellets	30	Medical scientist
	Incubate tubes	5	Medical scientist
	Load samples into well plate	15	Medical scientist
	Incubate plate	30	Medical scientist
	Measure absorbance	10	Medical scientist
	Calculate protein concentration	5	Medical scientist
**Spin Column Digestion**[a]	Sample normalization	20	Medical scientist
	Protein reduction with TCEP & Alkylation with CAA	7	Medical scientist
	Acidification with phosphoric acid	5	Medical scientist
	Mix sample with binding/wash buffer & Trap samples in S-Trap column	15	Medical scientist
	Centrifuge columns	2	Medical scientist
	Clean protein	7	Medical scientist
	Incubate and digest overnight	10	Medical scientist
	Elute peptides for analysis	20	Medical scientist
	Sample dry down	5	Medical scientist
**Liquid Chromatography with tandem mass spectrometry (LC-MS/MS)**[b]	Calibration	2	Medical scientist
	Loading gradients	10	Medical scientist
	Running gradients	0	Automated process
	Cleaning	5	Medical scientist
	Troubleshooting	10.375	Senior principal research Scientist
**Bioinformatics**	Download and batch results	8.5	Medical scientist (PhD)
	Data Analysis (includes data archiving)	75	Medical scientist (PhD)
	Re-Analysis	1.5	Medical scientist (PhD)

	Maintenance	12.92	Medical scientist
**Reporting**	Report drafting	65	Medical scientist (PhD)
	Report reviews	30	Principal Scientist
	Final report writing	37.5	Medical scientist (PhD)

a = Labor use per activity was estimated per batch (equivalent to 24 samples or 6 patients)

b = Labor use per activity was estimated per sample

c = Labor use per activity was estimated per patient (equivalent to 3 samples per patient)

Supplementary Table 3a: Unit costs for calculation of labor costs

Personnel type	Grade(s)	Salary Midpoint (weekly, 38h)	Rate per minute (Midpoint)	References
**Medical scientist trainee**	1-2	$1,007.00	$0.442	pp142 VPHS 2021-25 BR5-BR6 (FFPPOA 1/Dec/2022)
**Medical scientist**	1-2	$1,576.55	$0.691	pp142 VPHS 2021-25 RX1-RY7 (FFPPOA 1/Dec/2022)
**Medical scientist (Supervisor)**	3-4	$2,376.45	$1.042	pp142 VPHS 2021-25 RY9-RZ7 (FFPPOA 1/Dec/2022)
**Medical scientist (PhD)**	1-2	$1,709.95	$0.750	pp142 VPHS 2021-25 RX1-RY7 (FFPPOA 1/Dec/2022) with PhD Allowance of $133.40 p/w (PP 143)
**Senior principal research Scientist**	5	$3,357.00	$1.472	pp142 VPHS 2021-25 RZ8 (FFPPOA 1/Dec/2022)
**"Scientist Deputy Director /**	RZ9	$3,099.00	$1.359	pp142 VPHS 2021-25 RZ9 (FFPPOA 1/Dec/2022)
**Operations Manager / Business Manager"**	SA1	$3,465.90	$1.520	pp142 VPHS 2021-25 SA1 (FFPPOA 1/Dec/2022)
**Scientist Director**	SA2	$3,099.50	$1.359	pp142 VPHS 2021-25 SA2 (FFPPOA 1/Dec/2022)

Source: *Schedule 2: Rates of Pay and Allowances, pp142, MEDICAL SCIENTISTS, PHARMACISTS AND PSYCHOLOGISTS VICTORIAN PUBLIC SECTOR (SINGLE INTEREST EMPLOYERS) ENTERPRISE AGREEMENT 2021-2025 -http://msav.org.au/documents/member-documents/agreements-2/public-sector/*

Supplementary Table 3b: Medical Scientists – Classification descriptors

Personnel type	Grade	PSR Codes	Minimum criteria for classification
**Medical scientist trainee**	1-2	BR5-BR6	Trainee Scientists (Persons who are engaged in studies leading to the attainment of being eligible for Graduate Membership of the Australian Institute of Medical Scientists)
**Medical scientist**	1	RX1-RX7	A Scientist who holds or is qualified to hold the degree of Bachelor of Applied Science Honours or Bachelor of Science Honours
	2	RY4-RY7	Is employed on work which requires special knowledge or depth of experience, and/or requires the application of a level of performance worthy of additional remuneration;
**Medical scientist (Supervisor)**	3	RY9-RZ3	would normally have been qualified (as defined) for at least eight years and is engaged on specialised scientific work or work of a research or developmental nature
	4	RZ5-RZ7	a scientist who would normally have at least ten years' experience, utilizing advanced and specialised professional knowledge and experience
**Senior principal research Scientist (Medical Scientist Grade 5)**	5	RZ8	a Scientist who is appointed as a senior principal research Scientist and who is responsible for the coordination of scientific effort on major research programme(s)

**Scientist Deputy Director / Operations Manager / Business Manager**	-	RZ9	A scientist with more than 10 years' experience who is a recognized discipline leader within their health service
**Scientist Director**	-	SA1	A Scientist who is: appointed a Deputy Director, Operations Manager or Business Manager of a scientific department in a teaching hospital
**Principal Scientist**	-	SA2	a senior Scientist who is appointed as Director of a scientific or diagnostic Department (however titled) in a health service

Source: *Schedule 3: Classification descriptors and higher qualification allowances, Section 6 -Medical Scientist, MEDICAL SCIENTISTS, PHARMACISTS AND PSYCHOLOGISTS VICTORIAN PUBLIC SECTOR (SINGLE INTEREST EMPLOYERS) ENTERPRISE AGREEMENT 2021-2025 - http://msav.org.au/documents/member-documents/agreements-2/public-sector/*

Supplementary Table 4: Consumables for sample processing

Stage	Item	Estimate*	Unit	Range - Min	Range - Max	Cost	Currency	Catalogue Number	Pack size	Unit Cost ($AUD)	Cost Range - Min	Cost Range - Max	Source
**PBMCs isolation**	SepMate™-15 (IVD) columns	24	units			$2,449.0	AUD	Catalog # 85420	500	$4.898	$4.898	$6.870	StemCell
	Ficoll® Paque Plus gradient	108	mL			$345.00	AUD	GE17-1440-02	600	$0.575			Sigma-Aldrich
	Phosphate Buffered Saline (PBS)	156	mL	144	168	$ 67.00	AUD	D8537-1L	1000	$0.067	$0.197	$0.061	Sigma-Aldrich
	Eppendorf tubes lo-bind	24	units			$128.88	AUD	30108442	500	$0.258	$0.258	$0.321	Eppendorf
	Falcon tube	24	units			$266.00	AUD	CLS430053	500	$0.532	$0.322	$1.880	Sigma-Aldrich
	P200, P1000 Pipette tips	120	units			$230.34	AUD	Refer to list	960	$0.240	$0.235	$0.254	Eppendorf
**Protein quantification - Bicinchoninic acid (BCA) assay**	Pierce™ BCA Protein Assay Kit (ThermoFisher)	5	mL			$463.00	AUD	23225	1000	$0.463	$0.463	$0.592	Thermo Fisher Scientific
	Benzonase	2	µL			$146.00	AUD	E1014 (≥250 units/µL)	20	$7.300	$4.54	$7.300	Sigma-Aldrich
	Falcon tube	1	units			$266.00	AUD	CLS430053	500	$0.532	$0.322	$1.880	Sigma-Aldrich
	96 well plate	1	units			$861.00	AUD	15041	100	$8.610			Thermo Fisher Scientific
	Multi-Channel Pipette Reservoir	1	units			$115.98	USD†	P8025-1S	100	$1.736			Southern Labware
	P10, P20, P1000 Tips	60	units			$230.34	AUD	Refer to list	960	$0.240	$0.235	$0.254	Eppendorf
**Spin Column Digestion**	S-trap columns	24	units			$274.99	USD†	S-Trap™ micro columns (≤ 100 µg)	40	$10.288	$5.312	$9.999	ProtiFi
	SDS, 20% Solution	1200	µL	900	1800	$113.00	AUD	AM9820	250,000	$0.00045			Thermo Fisher Scientific

	Triethylammonium bicarbonate (TEAB)	4000	µL			$695.00	AUD	18597	500,000	$0.00139	$0.001	$0.0020	Sigma-Aldrich
	2-Chloroacetamide (CAA)	46.7	mg			$96.00	AUD	C0267	500,000	$0.00019	$0.00016	$0.00065	Sigma-Aldrich
	Tris-(2-Carboxyethyl)phosphine, Hydrochloride (TCEP)	25	µL			$322.00	AUD	77720	5,000	$0.06440			Thermo Fisher Scientific
	Phosphoric acid	324	µL			$140.00	AUD	1005732500	2500000	$0.00006	$0.00001	$0.00006	Sigma-Aldrich
	HPLC-grade water	5000	µL			$ 30.20	AUD	1153331000	1000000	$0.00003	$0.00003	$0.00003	Sigma-Aldrich
	Methanol	27000	µL			$173.70	USD†	A456-1	1000000	$0.00026	$0.00006	$0.00022	Thermo Fisher Scientific
	MS-grade Trypsin	48	µg			$222.00	AUD	20233	50000	$0.00444			Thermo Fisher Scientific
	Formic acid	2	µL			$274.00	AUD	85178	50000	$0.00548	$0.01	$0.02	Thermo Fisher Scientific
	Acetronitrile	500	µL	`		$886.00	AUD	85188	4000000	$0.00022			Thermo Fisher Scientific
	Eppendorf tubes lo-bind	48	units			$128.88	AUD	30108442	500	$0.258	$0.258	$0.321	Eppendorf
	P10, P200, P1000 tips	255	units			$230.34	AUD	Refer to list	960	$0.240	$0.235	$0.254	Eppendorf

**Estimates represent the volumes necessary to process a 24-sample batch (6 Patients) †AUD$1=$0.6682 USD as per the RBA exchange rate in July 2023*

Supplementary Table 5: Consumables for Liquid Chromatography with tandem mass spectrometry (LC-MS/MS)

Stage	Item	Estimate	Unit	Range - Min	Range - Max	Cost	Currency	Catalogue Number	Pack size	Unit Cost ($AUD)	Cost Range - Min	Cost Range - Max	Source
**Liquid Chromatography with tandem mass spectrometry (LC-MS/MS)**	Trap Columns (Acclaim™ PepMap™ 100 C18 NanoViper Trap Columns, 2/Pk)	140	units			$1,522.2	AUD	164946	2	$761.08	$450	$761.1	Thermo Fisher Scientific
	Main Columns (Acclaim™ PepMap™ 100 C18 Analytical Column, NanoViper)	12	units			$2,747.1	AUD	164570	1	$2,747.06	$1,700	$2,747	Thermo Fisher Scientific
	Acetonitrile (suitable for HPLC, gradient grade, ≥99.9%)	60	L			$188.00	AUD	34851-4L	4	$47.00	$32.1	$93.30	Sigma-Aldrich
	Isopropanol	20	L			$356.03	AUD	383920010	2.5	$142.41			Thermo Fisher Scientific
	10% DMSO	2	L			$447.00	AUD	34869-2L	1	$447.00	$875	$1,270	Sigma-Aldrich
	Pierce™ Formic Acid, LC-MS/MS Grade (10 x 1 mL)	120	mL			$218.00	AUD	28905	10	$21.80	$5.48	$21.80	Thermo Fisher Scientific
	nanoViper™ Fingertight Fittings	5	units			$499.22	AUD	6041.529	1	$499.22			Thermo Fisher Scientific
	Spray tips	12	units			$350.00	USD†		4	$130.944	$125	$200	Melbourne Mass Spectrometry and Proteomics Facility
	Pierce™ FlexMix™ Calibration Solution	60	mL			$507.00	AUD	A39239	10	$50.70			Thermo Fisher Scientific
	Performance Maintenance Kit	1	unit			$3910.3	AUD		1	$3910.3			Thermo Fisher Scientific
	Sample vials (Vrex™ Insert, 5mm Dia., 200µL, Clear 51, Flat Bottom, 1000/Pk)	14000	units			$262.92	AUD	AR0-4510-13	1000	$0.26	$0.25	$0.70	Phenomenex
	Pierce™ HeLa Protein Digest Standard (5x20 µg)	10	units			$882.00	AUD	88329	5	$176.40	$176.4	$227.	Thermo Fisher Scientific
	Nitrogen G-Size 7.2m3 (High Purity Grade, Compressed)	3	units	2	4	$290.31	AUD	034G	1	$290.31			BOC Australia

** Estimates represent the volumes necessary to run the entire facility per annum. The unit costs were divided by the estimated output per year (supplementary table 1a) to calculate the cost per sample. †AUD$1=$0.6682 USD as per the RBA exchange rate in July 2023*

Supplementary Table 6: List of equipment used by stage

**Stage**	Item	Catalogue Name	Price	Currency	Source	Unit	Useful Life (Yrs)	Annuity factor	Annual Cost	Annual Output*	Cost per sample ($AUD)
**PBMCs isolation**	Centrifuge for 1.5 mL tubes	MyFuge™ 12 mini centrifuge	448	USD†	Sigma-Aldrich	1	10	7.72	$58.02	3,942	$0.0220
	Centrifuge for 15 mL tubes	Medifuge™ Small Benchtop Centrifuge	3,517.19	AUD	Thermo Fisher Scientific	1	10	7.72	$455.49	3,942	$0.1155
	P200 Pipette	F1-ClipTip™ Variable Volume Single Channel Pipettes (20 to 200 µl)	374.92	AUD	Thermo Fisher Scientific	1	10	7.72	$48.55	3,942	$0.0123
	P1000 Pipette	F1-ClipTip™ Variable Volume Single Channel Pipettes (100 to 1000 µL)	374.92	AUD	Thermo Fisher Scientific	1	10	7.72	$48.55	3,942	$0.0123
**Protein quantification - Bicinchoninic acid (BCA) assay**	Multichannel pipette	Finnpipette™ Novus Multichannel Pipettes (5 to 50 µL)	$1,743.44	AUD	Thermo Fisher Scientific	1	10	7.72	$225.78	3,942	$0.0573
	Plate reader	Thermo Scientific Varioskan Flash 96	$8245	USD†	New Life Scientific	1	10	7.72	$1067.77	3,942	$0.4054
	Computer	MacBook Pro M2 16Gb	2,690	AUD	Apple	1	10	7.72	$348.37	3,942	$0.088
	Incubator (37 degrees)	ProSciTech, Mini bench top incubators, +5C to +65C (20L)	$2,473.90	AUD	ProSciTech	1	10	7.72	$320.38	3,942	$0.081
	Pipette	F1-ClipTip™ Variable Volume Single Channel Pipettes (100 to 1000 µL)	374.92	AUD	Thermo Fisher Scientific	1	10	7.72	$48.55	3,942	$0.012
**Spin Column Digestion**	Incubator (37 degrees)	ProSciTech, Mini bench top incubators, +5C to +65C (20L)	2,473.90	AUD	ProSciTech	1	10	7.72	$320.38	3,942	$0.081
	Speedvac	SpeedVac™ SRF110 Refrigerated Centrifugal Vacuum Concentrator	$14,066.58	USD†	Avantor®	1	10	7.72	$1821.69	3,942	$0.692
	Heatblock (99 degrees)	Touch Screen Dry Bath/Block Heater (1 Block)	989.67	AUD	Thermo Fisher Scientific	1	10	7.72	$128.17	3,942	$0.033

	Centrifuge for 15 mL tubes	Medifuge™ Small Benchtop Centrifuge	3,517.19	AUD	Thermo Fisher Scientific	1	10	7.72	$455.49	3,942	$0.1155
	Pipette	F1-ClipTip™ Variable Volume Single Channel Pipettes (100 to 1000 µL)	374.92	AUD	Thermo Fisher Scientific	1	10	7.72	$48.55	3,942	$0.012
**Liquid Chromatography with tandem mass spectrometry (LC-MS/MS)**	Orbitrap Exploris™ 480 Mass Spectrometer	Mass Spectrometer	$980,000	AUD	Thermo Fisher Scientific	1	10	7.72	$126914.48	3,942	$32.195
	Ultimate 3000 RSLC nanoHPLC	nanoHPLC	$120,000	AUD	Thermo Fisher Scientific	1	10	7.72	$15540.55	3,942	$3.942
	Service Support Plan - Orbitrap Exploris™ 480 Mass Spectrometer	Service Support Plan, Thermo Fisher Scientific	$31,698.50	AUD	Thermo Fisher Scientific	1	6	5.08	$6245.16	3,942	$1.584
	Service Support Plan-HPLC	Service Support Plan, Thermo Fisher Scientific	$12,689.50	AUD	Thermo Fisher Scientific	1	6	5.08	$2500.05	3,942	$0.634
	MiLiQ System	Milli-Q® IQ 7000 Ultrapure Water System	15,450.00	USD†	Sigma-Aldrich	1	10	7.72	$2000.85	3,942	$0.760
**Bioinformatics**	High Performance Computing - 32 CORES, 128 GB RAM	32 CORES, 128 GB RAM	15,000	AUD	Dell Workstation	1	5	4.33	$3464.62	39,420	$0.0878
	Spectronaut licensing		18,000	USD†	Bio21 Institute	1	10	7.72	$2331.08	3,942	$0.088
	Computer	MacBook Pro M2 16Gb	2,690	AUD	Apple	1	10	7.72	$348.37	3,942	$0.088
**Reporting**	Computer	MacBook Pro M2 16Gb	2,690	AUD	Apple	1	10	7.72	$348.37	3,942	$0.088
**Data Archiving**	Cloud storage	Research Data Platform - Mediaflux; $176 per TB per year	0.18	AUD	Resource Price Estimator: UoM Compute and Data Infrastructure	1 GB	1	1.00	$0.18	9.041	$7.95

**Annual output estimated as per estimated output per year (supplementary table 1). †AUD$1=$0.6682 USD as per the RBA exchange rate in July 2023*

Supplementary Table 7: Sample reception costs

Activity	Estimate	Cost Range (Min)	Cost Range (Max)	Source
**Blood Collection**	$ 28.68	$10.00	$75.00	UDN-Aus Sample Collection Agreements
**Fibroblast line establishment & culture**	$470.00	$425.00	$515.00	UDN-Aus Sample Collection Agreements
**Preparation & Referral of Fibroblast Cells**	$424.00	$375.00	$473.00	UDN-Aus Sample Collection Agreements
**Sample handling and processing**	$ 25.00	$12.00	$50.00	UDN-Aus Sample Collection Agreements
**Sample Transport/Shipping**	$ 39.00	$20.00	$60.00	UDN-Aus Sample Collection Agreements

PART 5: Probabilistic distribution

All cost inputs were fitted into a gamma distribution and entered the model as parameters of the probabilistic distribution. The model drew random values for each unit cost from the distributions, which were then multiplied by their respective resource use for each item of consumables, equipment, or labor task. The individual costs were finally aggregated to produce a cost per patient. This process was replicated 10,000 times using a MonteCarlo simulation. The estimates are presented as the mean values of the replicates, with their respective 95% confidence intervals.

Supplementary Table 8 shows the shape (α) and rate parameters (β) of the gamma distribution for each unit cost. These parameters were estimated via the method of moments, utilizing the mean **μ** and variance $\boldsymbol{\sigma^2}$ from each unit cost as follows:

$$\boldsymbol{\alpha} = \frac{\mu^2}{\sigma} \ , \ \boldsymbol{\beta} = \frac{\mu}{\alpha}$$

For the unit cost estimates where no range could be obtained (i.e., only one observation was recorded), the variance was made equal to the mean $\boldsymbol{\mu = \sigma^2}$.

Supplementary Table 8: Distributions used in the probabilistic analysis

Item	Estimate	Range	Distribution
**Consumables**			
SepMate™-15 (IVD) columns	$4.89800	4.9 - 6.87	Gamma α= 23.81, β= 0.23
Ficoll® Paque Plus gradient	$0.57500	-	Gamma α= 0.58, β= 1
Phosphate Buffered Saline (PBS)	$0.06700	0.2 - 0.06	Gamma α= 2, β= 0.05
Eppendorf tubes lo-bind	$0.25776	0.26 - 0.32	Gamma α= 57.98, β= 0
Falcon tube	$0.53200	0.32 - 1.88	Gamma α= 1.16, β= 0.78
P200, P1000 Pipette tips	$0.23994	0.24 - 0.25	Gamma α= 657.46, β= 0
Pierce™ BCA Protein Assay Kit (ThermoFisher)	$0.46300	0.46 - 0.59	Gamma α= 46.16, β= 0.01
Benzonase	$7.30000	4.54 - 7.3	Gamma α= 16.03, β= 0.4
Falcon tube	$0.53200	0.32 - 1.88	Gamma α= 1.16, β= 0.78
96 well plate	$8.61000	-	Gamma α= 8.61, β= 1
Multi-Channel Pipette Reservoir	$1.73564	-	Gamma α= 1.74, β= 1
P10, P20, P1000 Tips	$0.23994	0.24 - 0.25	Gamma α= 657.46, β= 0
Eppendorf tubes lo-bind	$0.25776	0.26 - 0.32	Gamma α= 57.98, β= 0
S-trap columns	$10.28806	-	Gamma α= 9.33, β= 0.91
SDS, 20% Solution	$0.00045	5.31 - 10	Gamma α= 0, β= 1
Triethylammonium bicarbonate (TEAB)	$0.00139	-	Gamma α= 19.9, β= 0
Chloroacetamide (CAA)	$0.00019	-	Gamma α= 1.47, β= 0
Tris-(2-Carboxyethyl)phosphine, Hydrochloride (TCEP)	$0.06440	-	Gamma α= 0.06, β= 1
Phosphoric acid	$0.00006	-	Gamma α= 2.49, β= 0
HPLC-grade water	$0.00003	-	Gamma α= 89.91, β= 0
Methanol	$0.00026	-	Gamma α= 2.76, β= 0
MS-grade Trypsin	$0.00444	-	Gamma α= 0, β= 1

Formic acid	$0.00548	-	Gamma α= 1.34, β= 0.01
Acetronitrile	$0.00022	0.01 - 0.02	Gamma α= 0, β= 1
Eppendorf tubes lo-bind	$0.25776	0.26 - 0.32	Gamma α= 57.98, β= 0
P10, P200, P1000 tips	$0.23994	0.24 - 0.25	Gamma α= 657.46, β= 0
Trap Columns (Acclaim™ PepMap™ 100 C18 NanoViper Trap Columns, 2/Pk)	$761.07500	450 - 761.08	Gamma α= 13.4, β= 49.07
Main Columns (Acclaim™ PepMap™ 100 C18 Analytical Column, NanoViper)	$2,747.06000	1700 - 2747.06	Gamma α= 15.74, β= 152.39
Acetronitrile (suitable for HPLC, gradient grade, ≥99.9%)	$47.00000	32.06 - 93.3	Gamma α= 3.24, β= 17.74
Isopropanol	$142.41200	-	Gamma α= 142.41, β= 1
10% DMSO	$447.00000	875 - 1270	Gamma α= 4.41, β= 196.09
Pierce™ Formic Acid, LC-MS/MS Grade (10 x 1 mL)	$21.80000	5.48 - 21.8	Gamma α= 3.01, β= 5.43
nanoViper™ Fingertight Fittings	$499.22000	-	Gamma α= 499.22, β= 1
Spray tips	$130.94375	125 - 200	Gamma α= 13.29, β= 11.44
Pierce™ FlexMix™ Calibration Solution	$50.70000	-	Gamma α= 50.7, β= 1
Calibration Tubing	$3,910.30000	-	Gamma α= 3910.3, β= 1
Sample vials (Vrex™ Insert, 5mm Dia., 200µL, Clear 51, Flat Bottom, 1000/Pk)	$0.26292	0.25 - 0.7	Gamma α= 2.49, β= 0.16
Pierce™ HeLa Protein Digest Standard (5 x 20 µg)	$176.40000	176.4 - 227	Gamma α= 43.77, β= 4.42
Nitrogen G-Size 7.2m3 (High Purity Grade, Compressed)	$290.31000	-	Gamma α= 290.31, β= 1
**Equipment**			
Centrifuge for 1.5 mL tubes	$448.00	-	Gamma α= 0.02, β= 1
Centrifuge for 15 mL tubes	$3,517.19	-	Gamma α= 0.12, β= 1
P200 Pipette	$374.92	-	Gamma α= 0.01, β= 1
P1000 Pipette	$374.92	-	Gamma α= 0.01, β= 1
Multichannel pipette	$1,743.44	-	Gamma α= 0.06, β= 1
Plate reader	$8,245.00	-	Gamma α= 0.41, β= 1
Computer	$2,690.00	-	Gamma α= 0.09, β= 1
Incubator (37 degrees)	$2,473.90	-	Gamma α= 0.08, β= 1
Speedvac	$14,066.58	-	Gamma α= 0.69, β= 1
Heatblock (99 degrees)	$989.67	-	Gamma α= 0.03, β= 1
Orbitrap Exploris™ 480 MS	$980,000.00	-	Gamma α= 32.2, β= 1
Ultimate 3000 RSLC nanoHPLC (Dionex Ultimate 3000)	$120,000.00	-	Gamma α= 3.94, β= 1

Maintenance Contract - Orbitrap Exploris™ 480 Mass Spectrometer	$31,698.50	-	Gamma α= 8.04, β= 1
Maintenance Contract - UltiMate™ 3000 RSLCnano System	$12,689.50	-	Gamma α= 3.22, β= 1
MiLiQ System	$15,450.00	-	Gamma α= 0.76, β= 1
High Performance Computing - 3 2 CORES, 128 GB RAM	$15,000.00	-	Gamma α= 0.13, β= 1
Spectronaut licencing	$18,000.00	-	Gamma α= 0.09, β= 1
Computer	$2,690.00	-	Gamma α= 0.09, β= 1
Cloud storage (estimate is per patient)	$0.18	-	Gamma α= 15.71, β= 1
**Labour (rate per minute)**			
Medical scientist trainee	$0.44	0.43 - 0.45	Gamma α=1844.07, β=0.0002
Medical scientist	$0.69	0.52 – 0.86	Gamma α=23.35, β=0.03
Medical scientist (Supervisor)	$1.04	0.88 - 1.20	Gamma α=63.6, β=0.01
Medical scientist (PhD)	$0.92	0.57 – 1.26	Gamma α=10.73, β=0.09
Senior principal research Scientist	$1.47	-	Gamma α=1.47, β=1
"Scientist Deputy Director /	$1.36	-	Gamma α=1.36, β=1
Operations Manager / Business Manager"	$1.52	-	Gamma α=1.52, β=1
Scientist Director	$1.36		Gamma α=1.36, β=1

PART 6: Analysis

Supplementary Table 9: Results of the scenario analysis

Annual patient throughput	Estimated Cost in AUD* (95% CI)	Difference from base case
**986 (Base Case)**	$897 (734-1111)	-
**100**	$3510 (3145-3922)	291%
**200**	$2058 (1811-2340)	129%
**300**	$1571 (1360-1824)	75%
**400**	$1330 (1135-1565)	48%
**500**	$1185 (1003-1413)	32%
**600**	$1088 (910-1312)	21%
**700**	$1020 (847-1244)	14%
**800**	$966 (799-1190)	8%
**900**	$926 (763-1138)	3%
**1000**	$892 (726-1102)	-1%
**1100**	$866 (706-1083)	-3%
**1200**	$846 (687-1066)	-6%
**1300**	$825 (664-1036)	-8%
**1400**	$812 (654-1027)	-9%
**1500**	$796 (638-1005)	-11%

**The estimates are presented as the mean values of 10,000 replicates using the probabilistic distribution of the input parameters.*

Supplementary Table 9: Results of the two-way sensitivity analysis

Annual patient throughput	Estimated Cost in AUD (Difference from base case estimate)		
	10% Shorter Bioinformatics Time	25% Less Consumables Costs	25% Less LC-MS/MS Equipment Costs
**986 (Base Case)**	$883 (-2%)	$854 (-5%)	$861 (-4%)
**100**	$3455 (285%)	$3342 (273%)	$3369 (276%)
**200**	$2026 (126%)	$1959 (118%)	$1975 (120%)
**300**	$1546 (72%)	$1496 (67%)	$1508 (68%)
**400**	$1309 (46%)	$1266 (41%)	$1277 (42%)
**500**	$1167 (30%)	$1128 (26%)	$1137 (27%)
**600**	$1071 (19%)	$1036 (15%)	$1044 (16%)
**700**	$1004 (12%)	$971 (8%)	$979 (9%)
**800**	$951 (6%)	$920 (3%)	$927 (3%)
**900**	$912 (2%)	$882 (-2%)	$889 (-1%)
**1000**	$878 (-2%)	$849 (-5%)	$856 (-5%)
**1100**	$852 (-5%)	$824 (-8%)	$831 (-7%)
**1200**	$833 (-7%)	$805 (-10%)	$812 (-9%)
**1300**	$812 (-9%)	$785 (-12%)	$792 (-12%)
**1400**	$799 (-11%)	$773 (-14%)	$779 (-13%)
**1500**	$784 (-13%)	$758 (-16%)	$764 (-15%)

Supplementary figure 1. Results of the two-way sensitivity analysis

The plot displays the results of joint changes in annual throughput levels and reductions in the acquisition costs of the LC-MS/MS platform or the bioinformatics processing time per patient. The green presents the variations in costs holding all other parameters constant (no reductions in costs or bioinformatics time). The dashed blue line presents the annual output estimated for the base case analysis (986 patients per annum).

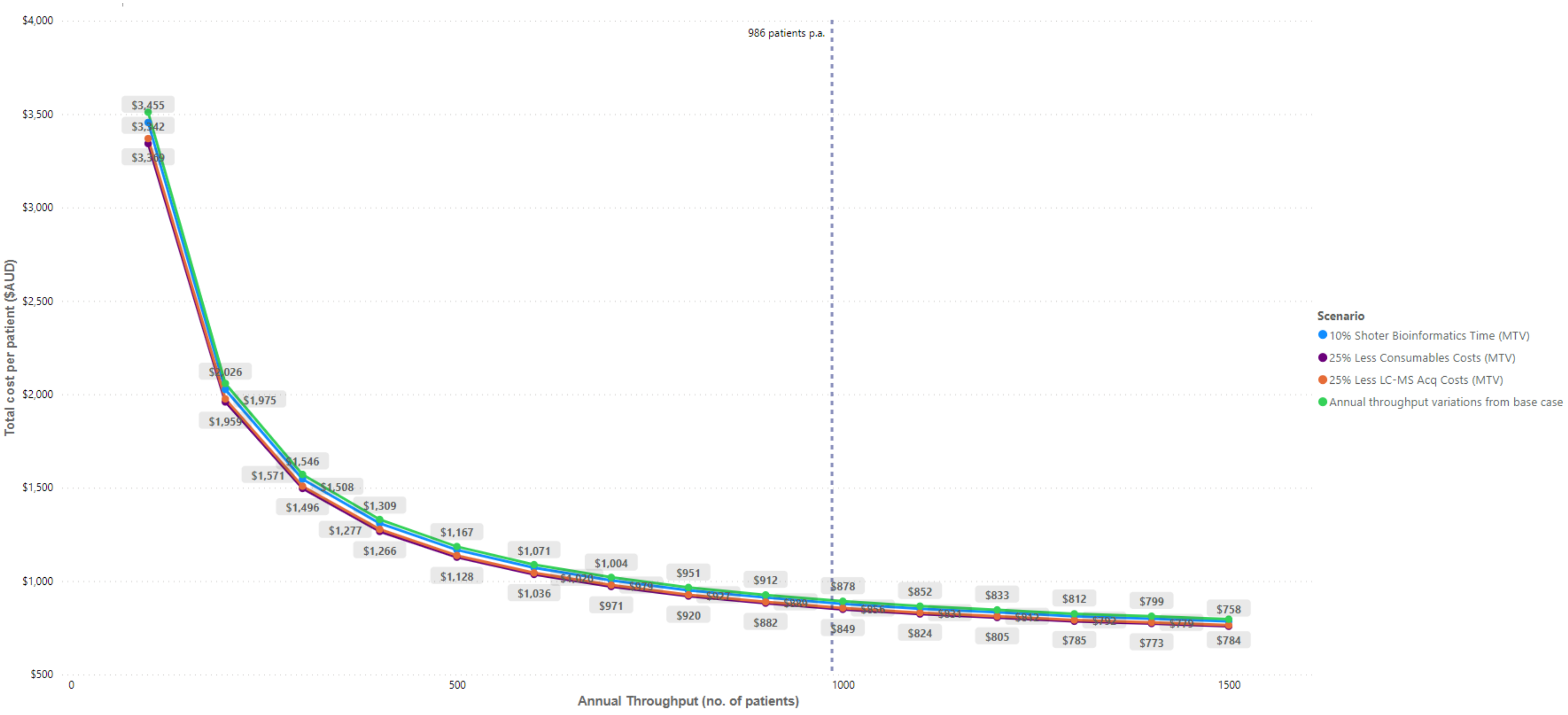

References

Barkovits, K., Linden, A., Galozzi, S., Schilde, L., Pacharra, S., Mollenhauer, B., Stoepel, N., Steinbach, S., May, C., Uszkoreit, J., Eisenacher, M., & Marcus, K. (2018). Characterization of Cerebrospinal Fluid via Data-Independent Acquisition Mass Spectrometry. *J Proteome Res*, *17*(10), 3418-3430. https://doi.org/10.1021/acs.jproteome.8b00308

Bruderer, R., Muntel, J., Müller, S., Bernhardt, O. M., Gandhi, T., Cominetti, O., Macron, C., Carayol, J., Rinner, O., Astrup, A., Saris, W. H. M., Hager, J., Valsesia, A., Dayon, L., & Reiter, L. (2019). Analysis of 1508 Plasma Samples by Capillary-Flow Data-Independent Acquisition Profiles Proteomics of Weight Loss and Maintenance. *Mol Cell Proteomics*, *18*(6), 1242-1254. https://doi.org/10.1074/mcp.RA118.001288

Hentschel, A., Czech, A., Münchberg, U., Freier, E., Schara-Schmidt, U., Sickmann, A., Reimann, J., & Roos, A. (2021). Protein signature of human skin fibroblasts allows the study of the molecular etiology of rare neurological diseases. *Orphanet J Rare Dis*, *16*(1), 73. https://doi.org/10.1186/s13023-020-01669-1

Muntel, J., Xuan, Y., Berger, S. T., Reiter, L., Bachur, R., Kentsis, A., & Steen, H. (2015). Advancing Urinary Protein Biomarker Discovery by Data-Independent Acquisition on a Quadrupole-Orbitrap Mass Spectrometer. *J Proteome Res*, *14*(11), 4752-4762. https://doi.org/10.1021/acs.jproteome.5b00826

Yang, L., Weng, S., Qian, X., Wang, M., & Ying, W. (2022). Strategy for Microscale Extraction and Proteome Profiling of Peripheral Blood Mononuclear Cells. *Anal Chem*, *94*(25), 8827-8832. https://doi.org/10.1021/acs.analchem.1c05365
